# Positive predictive value of ICD-10 codes for acute myocardial infarction in Japan: a validation study at a single center

**DOI:** 10.1186/s12913-018-3727-0

**Published:** 2018-11-26

**Authors:** Takashi Ando, Nobuhiro Ooba, Mayumi Mochizuki, Daisuke Koide, Koichi Kimura, Seitetz L. Lee, Soko Setoguchi, Kiyoshi Kubota

**Affiliations:** 1 Division of Evaluation and Analysis of Drug Information, Keio University Faculty of Pharmacy, Tokyo, Japan; 2 Department of Clinical Pharmacy, Nihon University School of Pharmacy, Chiba, Japan; 3 Department of Biostatistics & Bioinformatics Graduate School of Medicine The University of Tokyo, Tokyo, Japan; 4 Departments of Advanced Medical Science, The Institute of Medical Science, The University of Tokyo, Tokyo, Japan; 5 Department of Cardiovascular Medicine, University of Tokyo, Tokyo, Japan; 6 Duke Clinical Research Institute, Durham, NC USA; 7 NPO Drug Safety Research Unit Japan, Yushima 1-2-13-4F, Bunkyo-ku, Tokyo, 114-0002 Japan

**Keywords:** Acute myocardial infarction, Validation study, Positive predictive value; database, Case mix, Diagnosis procedure combination

## Abstract

**Background:**

In Japan, several large healthcare databases have become available for research since the early 2000’s. However, validation studies to examine the accuracy of these databases remain scarce. We conducted a validation study in order to estimate the positive predictive value (PPV) of local or ICD-10 codes for acute myocardial infarction (AMI) in Japanese claims. In particular, we examined whether the PPV differs between claims in the Diagnosis Procedure Combination case mix scheme (DPC claims) and in non-DPC claims.

**Methods:**

We selected a random sample of 200 patients from all patients hospitalized at a large tertiary-care university hospital between January 1, 2009 and December 31, 2011 who had an inpatient claim assigned a local or ICD-10 code for AMI. We used a standardized data abstraction form to collect the relevant information from an electronic medical records system. Abstracted information was then categorized by a single cardiologist as being either definite or not having AMI.

**Results:**

In a random sample of 200 patients, the average age was 67.7 years and the proportion of males was 78.0%. The PPV of the local or ICD-10 code for AMI was 82.5% in this sample of 200 patients. Further, of 178 patients who had an ICD-10 code for AMI based on any of the 7 types of condition codes in the DPC claims, the PPV was 89.3%, whereas of the 161 patients who had an ICD-10 code for AMI based on any of 3 major types of condition codes in the DPC claims, the PPV was 93.8%.

**Conclusion:**

The PPV of the local or ICD-10 code for AMI was high for inpatient claims in Japan. The PPV was even higher for the ICD-10 code for AMI for those patients who received AMI care through the DPC case mix scheme. The current study was conducted in a single center, suggesting that a multi-center study involving different types of hospitals is needed in the future. The accuracy of condition codes for DPC claims in Japan may also be worth examining for conditions other than AMI such as stroke.

## Background

Acute myocardial infarction (AMI) is one of the major causes of morbidity and mortality although its incidence varies between countries. For example, the incidence is much lower in Japan and other East Asian countries compared to the US and Europe [1]. Over the past two decades, the increased risk of AMI has been demonstrated to be associated with a variety of medications including non-steroidal anti-inflammatory drugs [2, 3], antidiabetic drugs [4, 5] and anti-human immunodeficiency virus drugs [6]. Administrative healthcare databases are increasingly used in observational studies enabling access to data for a large number of patients in a relatively short period of time and at a moderate cost [7]. Data indicating the occurrence of an outcome such as AMI should be accurate enough to make the study results reliable [8]. According to a systematic review on 30 validation studies of AMI published between 1984 and 2010 in North America and Europe, most studies assessed the International Classification of Disease and Related Health Problems 9th revision (ICD-9) code of AMI in hospitalized patients and found the positive predictive value (PPV) to be 93% or higher, and that the sensitivity and specificity were 86% or higher [9]. After 2010, along with further validation studies of AMI conducted in North America and Europe [10, 11], studies in Asian countries also emerged. In a study in Korea, the PPV of the ICD-10 codes of AMI in insurance claims ranged from 71.4 to 73.1% [12]. In Taiwan, the PPV of the ICD-9 codes of AMI in insurance claims was 88% [13].

In Japan, the claims database [14] and the electronic healthcare database [15] became available on a commercial basis after 2000. A large nationwide database (“NDB Japan”) was created in the late 2000’s and the provision of data for research commenced in 2011 [16–18].

In Japan, under a universal health insurance system, reimbursements have been made on a fee-for-service basis since 1961 [19, 20]. In 2003, following the introduction of the concept of “case mix”, the Diagnosis Procedures Combination (DPC) scheme was created for acute hospital care [20]. Differing from payment on a per case base in the Diagnosis Related Group (DRG) in the US, a DPC payment is made on a flat-rate per day basis as specified for each DPC classification [21]. About 5000 DPC classifications (in 2016) are defined by the combination of one disease group code and other elements [22]. The DPC is not a mandatory scheme that hospitals must follow. However by 2010, 1388 (18%) of a total 7587 hospitals in the country adopted the DPC reimbursement scheme. As these DPC hospitals are relatively large, in 2010 it was observed that 455,148 (50.4%) of a total 903,621 hospital beds for general and acute care in Japan were provided for by the DPC scheme [19]. The DPC scheme covers more than 90% of acute in-patient care for cancer, injuries and cardiovascular and other diseases [20].

As the linkage of claims data and other data sources, including hospital medical charts at the individual level, is currently strictly prohibited in Japan, any validation study is challenging. One of few possible validation studies available is to use the claims data of the individual hospitals.

We conducted a validation study to evaluate the validity of the condition codes of AMI using the past claims data in a hospital. In particular, we studied whether the validity varies between DPC and non-DPC (i.e., fee-for-service) claims.

## Methods

A cross-sectional study was conducted at a large tertiary-care university hospital using all the fee-for-service claims and DPC claims issued in electronic format during the study period between January 1, 2009 and December 31, 2011. Those claims data issued in the past were available because any given hospital would normally keep claims data for at least 5 years according to government requirements. As basic data to characterize the study hospital, we examined the administrative data for the total number of hospitalized patients per year and the proportion of those hospitalized in the cardiology ward. In addition, we examined the top 3 diagnoses of all hospitalized patients as well as those patients in the cardiology ward.

The DPC hospitals issue a DPC claim for reimbursement. In addition, they may also issue a fee-for-service claim for care of diseases not covered by the DPC. On some occasions, the DPC hospitals may also issue a fee-for-service claim for acute in-patient care. For example, when a patient dies within 24 h after admission or when the length of stay in hospital exceeds the maximum limit specified for each DPC classification then the cost is not reimbursed in the DPC scheme but rather a fee-for-service claim is issued (with or without a DPC claim). In the current study, we assembled the information in the past DPC and non-DPC claims data in the hospital during the study period and constructed a data set, which is presumably the same as the data in the claims database.

The NDB and other claims databases contain files compiled by assembling DPC and fee-for-service claims information issued by hospitals as Comma Separated Values (CSV) files. Coding of medical conditions differs between the DPC and the fee-for-service claims (Table 1). In a fee-for-service claim, the condition is specified by a 7-digit local condition code. Many codes may be recorded in a single fee-for-service claim and there is virtually no limit to the number of condition codes for any one claim. In addition, the primary diagnosis code, although available, is sometimes given to no, or multiple, condition codes. In the case of a DPC claim, two classes of condition codes are recorded [23]. One class (defined herein as the “class A” condition code) is a 6-digit disease group code. The disease group code is then used for the first 6 digits of a 14-digit DPC classification code. The other class (the “class B” condition code) consists of a maximum of 7 types of conditions that are recorded as a pair consisting of a 7-digit local condition code (as in the fee-for-service claim) and an ICD-10 code. Of the 7 types of class B condition codes, 3 are mandatory and always specified in every DPC claim (B-1 to B-3 in Table 1) [24]. One of these three (B-1 in Table 1) should belong to a disease group specified by the class A condition code.

**Table 1:** Condition codes in claims for DPC and non-DPC hospitalizations

Type of claims for hospitalization	Description
I. Claims for DPC hospitalization	
A. DPC code	A mandatory code with 14 digits. The first 6 digits represent a group of conditions to which B-1 below belongs, followed by the 8 digits indicating whether or not the patient had surgical operation, other procedures and other relevant conditions
B. ICD-10 codes^a^	ICD10 codes for 7 categories of conditions (B-1 to B-7 below)
B-1. Greatest-resource condition	A mandatory code for a condition responsible for the greatest use of resources
B-2. Trigger-for-hospitalization condition	A mandatory code for a condition that triggered hospitalization.
B-3. Main condition	A mandatory code for a condition given as the main condition in the discharge summary
B-4. Other condition	A code for a condition required to record when the DPC code indicates that the patient had a defined other relevant condition
B-5. Second greatest-resource condition	An optional code for a condition responsible for the second greatest use of resources
B-6. Comorbidities at the time of admission	A maximum of 4 codes for comorbidities that the patient had at the time of admission
B-7. Conditions occurring during the hospitalization	A maximum of 4 codes for conditions occurring during the hospitalization
II. Claims for non-DPC hospitalization	
Condition codes	Local condition codes with 7 digits. They are officially mapped to ICD-10 codes with a few exceptions. No limitation is set to the maximum number of conditions per claim.
Primary-condition flag	The flag indicating that the condition is the primary condition. The flag is often not given to any condition or given to two or more conditions

*DPC* Diagnosis Procedure Combination, *ICD-10* International Classification of Diseases and Related Health Problems 10th Revision

^a^The same condition code may be given to different types of conditions (e.g., the same ICD10 code may be given to all of B-1, B-2 and B-3)

In the claims in CSV-format, each single line pertains to one condition, one drug or one procedure, etc., and has a specific header. For example, the header “SY” is for a local condition code in the fee-for-service claim, “BU” for a DPC classification while “SB” is used for a pair of local and ICD-10 codes in the DPC claim. The NDB and other claims databases are constructed by assembling claims information by reference to a specific header. By similarly assembling the information of the past claims data at the study hospital, a data set, which is considered to be the same as the data in the claims database, was constructed and its accuracy was evaluated.

We identified 299 patients with DPC claims assigned an ICD-10 code of I21, I22 or I23 (AMI) as part of the 7 types of class B condition codes (in “SB” data) and/or fee-for-service claims with 7-digit local condition codes for AMI (in “SY” data) during the study period. When a patient was hospitalized twice or more due to AMI during the study period, we only used claims for the first hospitalization in this study. From the 299 patients with AMI claims, we selected a random sample of 200 patients. To select 200, we arranged 299 with AMI in ascending order according to a random variable assigned to each of them and simply selected the first 200. In order to validate the condition code in the claims, information was abstracted from the electronic medical records system using a standardized data abstraction form employed in a validation study of AMI conducted in the Mini-Sentinel program in the US [10]. Referencing the abstracted information and, if required, the original data in the electronic medical records system, a cardiologist then categorized each case as either “definite AMI” vs “no AMI” while remaining blinded to the diagnosis code contained in the claims. As in a previous validation study of AMI [10], definite AMI was defined as the condition satisfying one of the 5 criteria: (i) detection of the rise and/or fall of cardiac biomarkers accompanied by ischemic symptoms, electrocardiographic (ECG) change or imaging evidence; (ii) sudden unexpected cardiac death; (iii) percutaneous coronary intervention (PCI) related MI (iv) coronary artery bypass grafting (CABG) related MI and (v) postmortem pathologic finding. In the current study no case was classified as “probable MI” [10].

### Statistical analysis

We estimated the PPV and its 95% exact binomial confidence interval for a condition code of AMI: (i) in any claim; (ii) in any type of class B condition code (B1 to B7 in Table 1) in the DPC claim, and (iii) in one or more of the 3 major types (B1 to B3 in Table 1) in the DPC claim. We did not, however, estimate measures of validity other than the PPV such as the sensitivity, specificity and negative predictive values because: a) good registration data for MI covering the whole hospital was unavailable, and b) the limited resources precluded a chart review of all the patients in the hospital or a large enough random sample of the hospitalized patients during the study period. The study was approved by the ethics committee of University of Tokyo (Ref No. 3705). The statistical analysis was done with SAS V.9.4 (SAS Institute).

## Results

During the study period (2009–2011), on average, a total of 25,519 patients were hospitalized per year of which 1660 (6.5%) were in the cardiology ward in the study hospital. The first and second most frequent disease groups amongst all hospitalized patients were “liver cancer” and “angina/chronic ischemic cardiac disease” in each of the 3 years, while the third most frequent disease group was “lung cancer” in 2009 and “cataract” in 2010 and 2011. The first, second and third most frequent disease groups in the cardiology ward were “angina/chronic ischemic cardiac disease”, “heart failure” and “tachyarrhythmia” in each of the 3 years. During the study period, AMI ranked 65th to 73rd of a total of 419 to 438 disease groups across the whole hospital and ranked 5th (following “bradyarrhythmia”) of 66 to 74 disease groups in the cardiology ward.

The demographic and other characteristics of a random sample of 200 hospitalized patients with condition codes of AMI in any claims are shown in Table 2. The average age was 67.7 years and the proportion of males was 78.0%. About one third of 200 patients were admitted in each year during the study period between 2009 and 2011. Of the sample of 200 hospitalized patients, the ICD-10 code for 104 patients (i.e., 52%) was “Acute transmural myocardial infarction of anterior (I21.0) or inferior (I21.1) wall” whereas the ICD-10 code was “Acute myocardial infarction, unspecified (I21.9)” for 61 patients (i.e., 30.5%). The code for “Subsequent myocardial infarction” (I22) was not used in any claim for the sample of 200 patients. As to therapeutic intervention for AMI, 140 (70%) of 200 patients undertook PCI, thrombolytic therapy or CABG.

**Table 2:** A random sample of 200 hospitalized patients with an ICD10 diagnosis code of AMI

	True AMI	No AMI	Total
	*N* = 165	*N* = 35	*N* = 200
Age (SD) years	67.3 (12.6)	69.4 (9.0)	67.7 (12.0)
Male, N (%)	126 (76.4)	30 (85.7)	156 (78.0)
Year of admission, N (%)			
2009	61(37.0)	12 (34.3)	73 (36.5)
2010	51 (30.9)	11 (31.4)	62 (31.0)
2011	53 (32.1)	12 (34.3)	65 (32.5)
ICD10 codes in claims, N (%)^a^			
I21.0 Acute transmural myocardial infarction of anterior wall	58 (35.2)	1 (2.9)	59 (29.5)
I21.1 Acute transmural myocardial infarction of inferior wall	45 (27.3)	0 (0)	45 (22.5)
I21.2 Acute transmural myocardial infarction of other sites	18 (10.9)	0 (0)	18 (9.0)
I21.3 Acute transmural myocardial infarction of unspecified site	1 (0.6)	0 (0)	1 (0.5)
I21.4 Acute subendocardial myocardial infarction	0 (0)	1 (2.9)	1 (0.5)
I21.9 Acute myocardial infarction, Unspecified	43 (26.1)	18 (51.4)	61 (30.5)
Procedures for AMI in claims, N (%)	131 (79.4)^b^	9 (25.7)^b^	140 (70.0)^b^
Percutaneous coronary intervention (PCI)	119 (72.1)	9 (25.7)	128 (64.0)
Thrombolytic therapy(TT)	3 (1.8)	1 (2.9)	4 (2.0)
Coronary artery bypass graft(CABG)	14 (8.5)	0 (0)	14 (7.0)

*ICD-10* International Classification of Diseases and Related Health Problems 10th Revision, *AMI* acute myocardial infarction, *SD* Standard Deviation

^a^No patient had the code for “Subsequent myocardial infarction” (I22)

^b^The number (%) of patients who had either PCI, TT or CABG

Table 3 shows the PPV for the condition codes for the different kinds of claims. The PPV was 82.5% in the 200 patients assigned an AMI condition code in any claim. The PPV was 89.3% for the 178 patients with a code of AMI in the DPC claims, and 93.8% for the 161 patients with a condition code of AMI as type B-1, B-2 or B-3 in the DPC claims.

**Table 3:** PPV of condition codes for AMI in DPC and non-DPC claims

Definition	N	True AMI	PPV (95% confidence interval)^a^
Condition code for AMI in DPC or non-DPC claims	200	165	82.5 (76.5–87.5)%
Condition code for AMI in DPC claims	178	159	89.3 (83.8–93.4)%
Condition code for AMI in non-DPC claims only	22	6	27.3 (10.7–50.2)%
Condition code for AMI in either of type B-1, B-2 or B-3 in DPC claims^b^	161	151	93.8 (88.9–97.0)%
Condition code for AMI in either of type B-5, B-6 or B-7 but not in B-1 B-2 or B-3 in DPC claims ^bc^	17	8	47.1 (23.0–72.2)%

*PPV* positive predictive value, *AMI* acute myocardial infarction, *DPC* Diagnosis Procedure Combination

^a^Exact binomial confidence interval

^b^See Table 1 for B-1, B-2, B-3, B-5, B-6 and B-7

^c^No patient had AMI as “other condition” (B-4)

## Discussion

In the current study, the PPV of the condition code of AMI in the claims of hospitalized patients was high. The PPV was even higher when restricted to patients with an AMI code in the DPC claims, particularly when the condition was recorded as one of the 3 major types of class B condition codes (B-1, B-2 and B-3 in Table 1).

So far, only a few validation studies of the information contained in the databases of claims (and other healthcare data) have been conducted in Japan [25–29]. One reason for this scarcity may be that database study is still relatively new to clinical studies in Japan. In one of the recent studies in Japan [29], 23 of 315 patients randomly selected from 4 DPC hospitals were found to have AMI in chart review and the PPV, sensitivity, and specificity of an ICD-10 code of AMI were 92.3, 52.2 and 99.7%, respectively.

We found that the PPV was higher in the DPC claims as compared to the fee-for-service claims. This observation may be relevant to the difference of the PPV of AMI in the studies conducted in Asian countries [12, 13]. For example, Taiwan introduced the DRG in 2010 for a wide variety of diseases including AMI [30] while the fee-for-service reimbursement is currently used for AMI in Korea where the use of the DRG-based reimbursement is limited to seven different diseases [31]. This difference of the reimbursement scheme might at least in part explain why the PPV in the study conducted in Korea [12] was lower than that in Taiwan [13].

In the current study, we found no patients who were hospitalized due to I22 (subsequent myocardial infarction) which is defined as “infarction of any myocardial site, occurring within 4 weeks (28 days) from onset of a previous infarction” [32]. The reason why none of the hospitalized patients had the diagnosis code of I22 was not clear. However, one of the likely reasons is that the concept of “subsequent myocardial infarction” has not been widely accepted among Japanese physicians as they normally diagnose AMI irrespective of whether or not the infarction has occurred within 4 weeks from the onset of a previous infarction.

The main limitation of the current study is that the study is conducted at a single hospital. Thus, the PPV estimates in this study should be interpreted carefully as the results may not necessarily be generalized to other hospitals or to the claims databases. Another limitation was that the chart review was conducted by only one cardiologist so that inter-rater agreement could not be assessed.

The strength of this study is that we compared the PPV of the condition code for AMI between DPC and non-DPC (fee-for-service) claims and demonstrated that the PPV of the condition code in DPC claims was higher. This observation was expected because the condition code in a DPC claim is carefully selected by a physician at the hospital. We believe that the results in this study suggest that the use of condition codes in DPC claims may be further elaborated against patient’s charts in validation studies of conditions other than AMI such as stroke for pharmacoepidemiology and other studies.

## Conclusion

In this study conducted between January 1, 2009 and December 31, 2011, we found that the PPV of the condition code of AMI in the claims at a large tertiary hospital was high. When restricted to DPC claims, the PPV was even higher. As the study was conducted at a single hospital, more studies for AMI, including a multi-center study involving different types of hospitals, are needed in the future. The accuracy of condition codes in DPC claims in Japan may also be worth examining for conditions other than AMI such as stroke in the future.

## Abbreviations

AMI: acute myocardial infarction
DPC: Diagnosis procedure combination
DRG: Diagnosis Related Group
ICD: International Classification of Disease and Related Health Problems
PPV: Positive predictive value
